# Adenoviral neutral endopeptidase gene delivery in combination with paclitaxel for the treatment of prostate cancer

**DOI:** 10.3892/ijo.2012.1586

**Published:** 2012-08-08

**Authors:** KATSUYUKI IIDA, RONG ZHENG, RUOQIAN SHEN, DAVID M. NANUS

**Affiliations:** 1 Genitourinary Oncology Research Laboratory, Department of Medicine, Weill Cornell Medical College and Weill Cornell Cancer Center;; 2 Division of Hematology and Medical Oncology, Department of Medicine; 3 Department of Urology, Weill Cornell Medical College, New York, NY, USA

**Keywords:** adenovirus, gene therapy, neutral endopeptidase, prostate cancer

## Abstract

Neutral endopeptidase (NEP) is a cell-surface peptidase that inhibits prostate cancer cell growth partly via inhibition of Akt kinase. We investigated the antitumor effects of an adenovirus gene delivery system (AdNEP) to restore NEP expression in DU145 prostate cancer cells in combination with paclitaxel chemotherapy. DU145 cells were infected with adenovirus expressing NEP or LacZ, treated with paclitaxel, and assessed for cell viability, Akt activation and induction of apoptosis. Athymic mice with established DU145 xenografts were injected intratumorally with AdNEP or AdLacZ and intraperitoneally with paclitaxel and monitored for tumor growth over 28 days. Compared to AdLacZ plus paclitaxel, AdNEP plus paclitaxel significantly inhibited DU145 cell growth and increased apoptosis as determined by increased caspase-3 and PARP-1 proteolytic fragments. In a xenograft model, tumor volume was reduced in mice treated with AdNEP plus paclitaxel (122.85±89.5 mm^3^; P<0.01) compared with mice treated with AdNEP plus saline (653.9±230.3 mm^3^), AdLacZ plus paclitaxel (575.9±176.6 mm^3^) or AdLacZ plus saline (920.2±238.2 mm^3^). In conclusion, these data suggest that NEP can augment taxane-induced apoptosis through inhibition of Akt/Bad signaling, and that the combination of NEP plus paclitaxel may be an effective strategy to inhibit castration-resistant prostate cancer growth.

## Introduction

Neutral endopeptidase (NEP, CD10) is a 90–110 kDa zinc dependent metallopeptidase that cleaves peptide bonds on the amino side of hydrophobic amino acids and inactivates a variety of bioactive peptides including atrial natriuretic factor, substance P, bradykinin, oxytocin, Leu- and Met-enkephalins, neurotensin, bombesin, endothelin-1, bombesin-like peptides and amyloid-β (1,2). Numerous tissues normally express NEP, including epithelial cells of the prostate, kidney, intestine, adrenal glands and lung. NEP expression is decreased or absent in nearly 50% of primary and metastatic prostate cancers (3–5). This loss can result from methylation of the NEP promoter or may occur after androgen withdrawal because NEP transcription is regulated by androgen (3,5,6). Replacement of NEP using either exogenous recombinant NEP or overexpression of NEP at the cell-surface using an NEP expression vector inhibits PC cell growth, cell migration and tumorigenicity (7,8). NEP actions have been demonstrated to result from catalytic inactivation of NEP substrates such as bombesin, endothelin-1 and basic fibroblast growth factor (bFGF), and via protein-protein interaction of NEP’s cytoplasmic domain with a variety of proteins, including ezrin/radixin/moesin proteins, Lyn kinase, and the phosphatase and tensin homolog (PTEN) protein (7,9,10). NEP recruits endogenous PTEN to the cell membrane, leading to prolonged PTEN protein stability and increased PTEN phosphatase activity, resulting in constitutive downregulation of Akt activity. NEP also indirectly inhibits Akt phosphorylation by catalytically inactivating peptides that normally activate the insulin growth factor-1 receptor (IGF-1R) leading to Akt phosphorylation. Thus, NEP regulates Akt via both catalytic-dependent and -independent pathways. In this regard, loss of NEP together with increased Akt phosphorylation in primary prostate cancers predicts a significantly shorter time to biochemical relapse in patients who have undergone radical prostatectomy (11).

We previously reported that recombinant NEP augments chemosensitivity of prostate cancer cells *in vitro* by promoting PKC δ-mediated mitochondrial apoptosis as determined by cytochrome-c release and caspase-9 activation (12). Taxanes, such as paclitaxel and taxotere, are active anticancer drugs that are currently used to treat advanced prostate cancer (13). The predominant mode of action of paclitaxel is the binding to β-tubulin, stabilizing the microtubule, and preventing its depolymerization (14). Recent studies suggest that activated Akt contributes to paclitaxel-induced resistance and thus inhibition of Akt may synergistically increase paclitaxel sensitivity (15,16). In the current study, we examined the combined antitumor effects on DU145 castration resistant prostate cancer cells of treatment with an adenovirus expressing NEP that inhibits Akt activation with paclitaxel.

## Materials and methods

### Cells and reagents

DU145 human PC cells (ATCC, Manassas, VA) were grown in RPMI-1640 medium supplemented with 2 mM glutamine, 1% non-essential amino acids, 100 units/ml streptomycin and penicillin, and 10% fetal calf serum (FCS). Antibodies used include anti-NEP antibody (NCL-CD10-270, Novocastra Laboratories Ltd., Newcastle upon Tyne, UK), anti-phospho-Akt antibody (Ser473, Cell Signaling Technology, Inc., Beverly, MA), anti-Akt antibody (Cell Signaling Technology, Inc.), anti-PTEN antibody (A2B1, Santa Cruz Biotechnology, Inc.), anti-phospho-BAD antibody (Ser136, Cell Signaling Technology, Inc.), anti-BAD antibody (Cell Signaling Technology, Inc.), caspase-3 antibody (Cell Signaling Technology, Inc.), PARP antibody (Cell Signaling Technology, Inc.), and anti-β-actin antibody (Sigma-Aldrich, St. Louis, MO).

### Adenovirus vector production and transduction

cDNA containing the entire full-length human NEP was used to construct and produce recombinant AdNEP plasmid (AdEasy™ Adenoviral Vector System, Stratagene). Adenovirus vectors were amplified in 293 cells and purified by cesium chloride density gradient ultracentrifugation. The AdLacZ vector was used as a negative control.

### NEP enzyme activity assay

NEP-specific enzyme activities was assessed as described using Suc-Ala-Ala-Phe-pNA (Bachem Bioscience Inc., Philadelphia, PA, USA) as substrate (3). Specific activities were expressed as picomoles per microgram of protein per minute and represent an average of two separate measurements performed in duplicate on separate occasions.

### Cell counts and cell viability assays

Following incubation overnight in T25 flask, cells were infected with AdNEP or AdLacZ at the indicated concentrations. Total cell numbers in three independent flasks in each group were counted using a hemocytometer, and the mean value of four fields was recorded. Cell viability was assessed by trypan blue, which was added to cell cultures at a ratio 1:1 and left for 10 min, and cells counted using a hemocytometer. The ratios of viable and dead cells were determined. For all experiments, data presented are representative of experiments performed at least three times in triplicate or quadruplicate.

### TUNEL assay

Apoptosis was determined in adherent DU145 cells cultured in a chamber slide or deparaffinized DU145 tumor tissue sections by Terminal deoxynucleotidyl transferase (TdT) mediated d-Uridine Tri Phosphate nick end labeling (TUNEL) technique using the In Situ Cell Death Detection kit, POD (Roche, Germany) according to the recommendations of the manufacturer. Quantitative evaluation of apoptotic cells was done by counting the TUNEL positive cells among those cells under light microscopy (×1000). The apoptotic index was expressed as TUNEL-positive cells per 100 cells.

### Immunoblotting

Total cell lysate preparation and immuno-blotting were performed as described (7,10). Proteins were visualized using enhanced chemiluminescence (Amersham Biosciences).

### Xenograft model

Under an IACUC approved protocol, DU145 cells (5×10^6^ cells) were inoculated subcutaneously into the flanks of 60 athymic male mice (Taconic, Hudson, NY, USA). When tumors reached a size of 4–6 mm in diameter, mice were randomly divided into 5 groups and treated with intratumor injection into the center and periphery of each mass on days 1 and 15, as follows: Group 1, 100 μl of saline (control); Groups 2 and 4, 100 μl of saline containing 1×10^8^ pfu of Ad-LacZ; Groups 3 and 5, 100 μl of saline containing 1×10^8^ pfu of Ad-NEP. Groups 4 and 5 also received paclitaxel administered i.p. at dose 10 mg/kg, daily from days 2 to 4 and from days 16 to 18 (17). Tumor volume was calculated as [0.52 × (W × W × L)] where W, minor axis and L, major axis and was measured every 3 days until day 28 post-adenoviral infection. Two animals were sacrificed on day 6 and the remainder on day 28 and the tumors dissected, weighed and collected for further analysis.

### Immunohistochemisty

Paraffin-embedded tissue sections were stained with anti-NEP antibody (NCL) at 1:80 dilution, anti-PTEN antibody at 1:100 dilution, anti-phospho-Akt antibody at 1:100 dilution using the Dako Envision + System HRP (Dako Cytomation, Carpinteria, CA) according to the manufacturer’s instructions.

### Statistical analysis

All analyses were done using Statview 5.0 (SAS Institute, Inc., Cary, NC). Results were expressed as the mean ± SE for three independent measurements. Analysis of variance was used for analysis of continuous data followed by Fisher’s protected least significant difference for post hoc analyses. Differences with a P<0.05 were determined as statistically significant.

## Results

### Adenoviral NEP expression in DU145 cells

**Figure 1:**
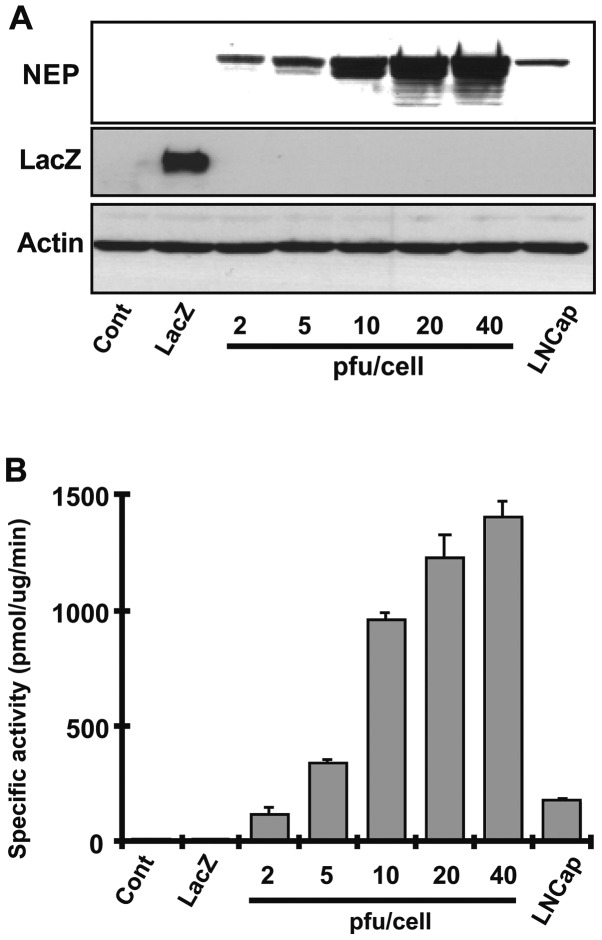
DU145 cells infected with AdNEP express catalytically active NEP protein. (A) Total cell lysates from DU145 cells infected with AdNEP or AdLacZ for 72 h at the indicated pfu/cell concentration were analyzed by immunoblotting using anti-NEP mouse monoclonal antibody NCL-CD10. Total cell lysate from LNCaP cells was used as positive control for NEP expression. Note dose-dependent increase in NEP protein in cells infected with AdNEP. Cells infected with AdLacZ at 10 pfu/cell expressed LacZ protein. (B) NEP-specific enzyme activity increased in DU145 cells infected with AdNEP for 72 h in a dose-dependent manner.

**Figure 2:**
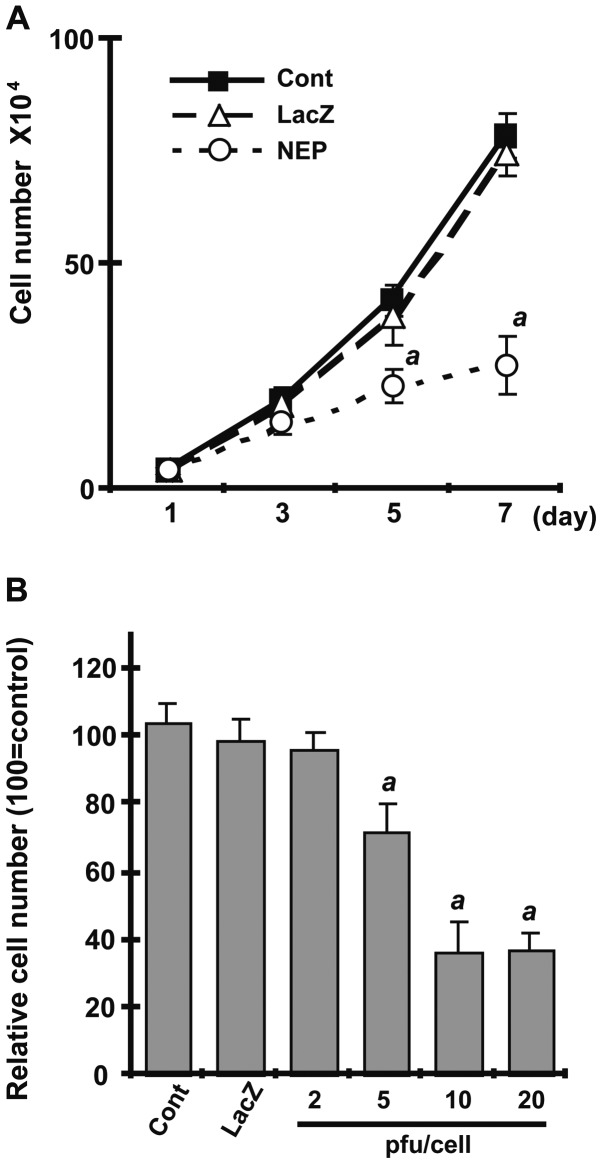
AdNEP expression inhibits DU145 cell growth. (A) Cells infected with AdNEP or AdLacZ at 10 pfu/cell, or uninfected cells were plated in T25 flask at 1×10^4^ cells per flask in triplicate and total cell numbers were counted at the indicated times. (B) Relative values of the number of cells infected with AdNEP at increasing concentrations compared to cells treated with AdLacZ at 10 pfu/cell. Cell growth was inhibited at 7 days in an adenovirus dose-dependent manner. Values are represented as mean ± SE (n=4). (a, P<0.01 compared with AdLacZ treated cells).

Adenoviral vectors expressing NEP or LacZ protein as control were introduced into DU145 cells at a pfu/cell concentration ranging from 2 to 40 and NEP protein levels and enzymatic activity were measured after 72 h. NEP protein expression and NEP specific enzyme activity increased in a pfu/cell dose-dependent manner (Fig. 1A and B). NEP activity in DU145 cells infected with AdNEP at 10 pfu/cell achieved an enzyme activity of 962.36±24.5 pmol/mg/min, a 10-fold higher specific activity than that achieved in DU145 infected lentiviral vector encoding NEP (96.57±10.2 pmol/mg/min) (18). NEP activity in uninfected DU145 cells or in DU145 cells infected with AdLacZ was 10.06±2.8 pmol/mg/min and 8.05±2.2 pmol/mg/min, respectively. We next examined the effect of NEP expression on cell growth, comparing uninfected DU145 cells with cells infected with AdNEP or AdLacZ at 10 pfu/cell. As shown in Fig. 2A, AdNEP significantly inhibited DU145 cell growth (P<0.01) compared with cells infected with AdLacZ or uninfected DU145 cells. Growth inhibition was dose-dependent (Fig. 2B). Together, these results show that adenoviral vectors can transduce catalytically active NEP protein into DU145 cells resulting in high levels of NEP catalytic activity and inhibition of cell growth.

### Adenoviral NEP infection in combination with paclitaxel

**Figure 3:**
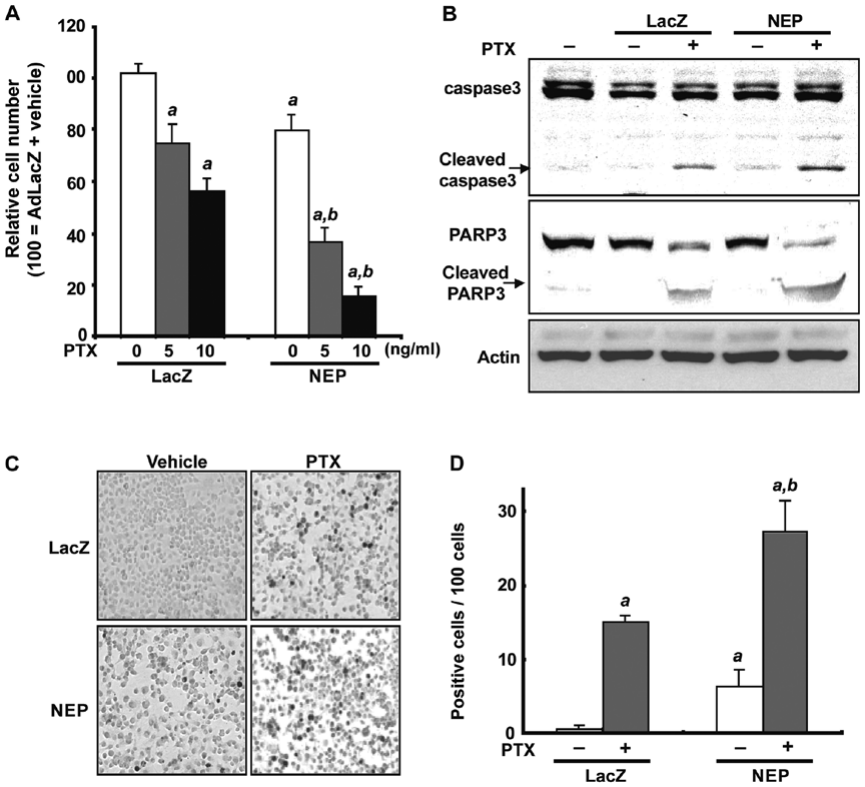
AdNEP infection augments paclitaxel induced apoptosis in DU145 cells. (A) Relative viable cell number of cells infected with AdNEP or AdLacZ in combination with paclitaxel. Following infection with AdNEP or AdLacZ at 10 pfu/cell, cells were treated with paclitaxel at 5 or 10 ng/ml, or vehicle for 48 h and counted at 5 days. Combination therapy with AdNEP plus paclitaxel significantly decreased cell viability. (B) Total cell lysates from DU145 cells treated as indicated and described above were separated by SDS-PAGE and immunoblotted with mAbs to caspase-3 and PARP-1. Infection with AdNEP increased caspase-3 and PARP-1 cleavage products compared with paclitaxel treatment of control cells infected with AdLacZ. (C) Representative immunostain of cells treated with AdLacZ or AdNEP plus paclitaxel. (D) Apoptotic index was calculated as the number of positive cells x 100/total number of cells counted under ×400 magnification in 10 randomly selected areas in each sample. a, P<0.01 compared with AdLacZ plus vehicle treated cells. b, P<0.01 compared with AdLacZ plus paclitaxel treated cells.

To assess the effect of NEP overexpression alone and in combination with paclitaxel, DU145 cells were infected with AdNEP or AdLacZ at 10 pfu/cell for 72 h and treated with paclitaxel at concentrations of 5 or 10 ng/ml for 48 h. As expected, paclitaxel significantly inhibited cell viability in cells infected with AdLacZ compared to control cells treated with vehicle with a relative viable cell number of 75.0±7.8% (5 ng/ml paclitaxel) or 56.4±4.8% (10 ng/ml paclitaxel) (p<0.01; Fig. 3A). In DU145 cells infected with AdNEP, paclitaxel treatment at 5 and 10 ng/ml resulted in decreased cell viability with a relative cell viability ratio of 36.7±5.3% and 15.5±3.8%, respectively, which was statistically significant compared to either control cells or cells infected with AdLacZ (p<0.01; Fig. 3A). Measurement of the AdNEP effect on paclitaxel-induced apoptosis was assessed by examining cleavage of caspase-3 and Poly-ADP-ribose polymerase 1 (PARP-1) and counting the number of cells undergoing apoptosis using a TUNEL assay. As shown in Fig. 3B, proteolytic fragments of caspase-3 and PARP-1 were evident at 48 h following paclitaxel treatment in DU145 cells infected with AdLacZ, which were increased in cells infected with AdNEP. The number of TUNEL-positive cells per 100 cells was significantly higher in DU145 cells treated with AdNEP plus paclitaxel (28.3±4.16) compared with cells treated with AdLacZ plus paclitaxel (16.3±1.52) or AdNEP plus vehicle (6.3±2.30) (P<0.01, Fig. 3C and D). These data demonstrate that NEP overexpression using AdNEP significantly accelerates paclitaxel-induced apoptosis in DU145 cells.

### Effects of AdNEP on Akt signaling and BAD expression

**Figure 4:**
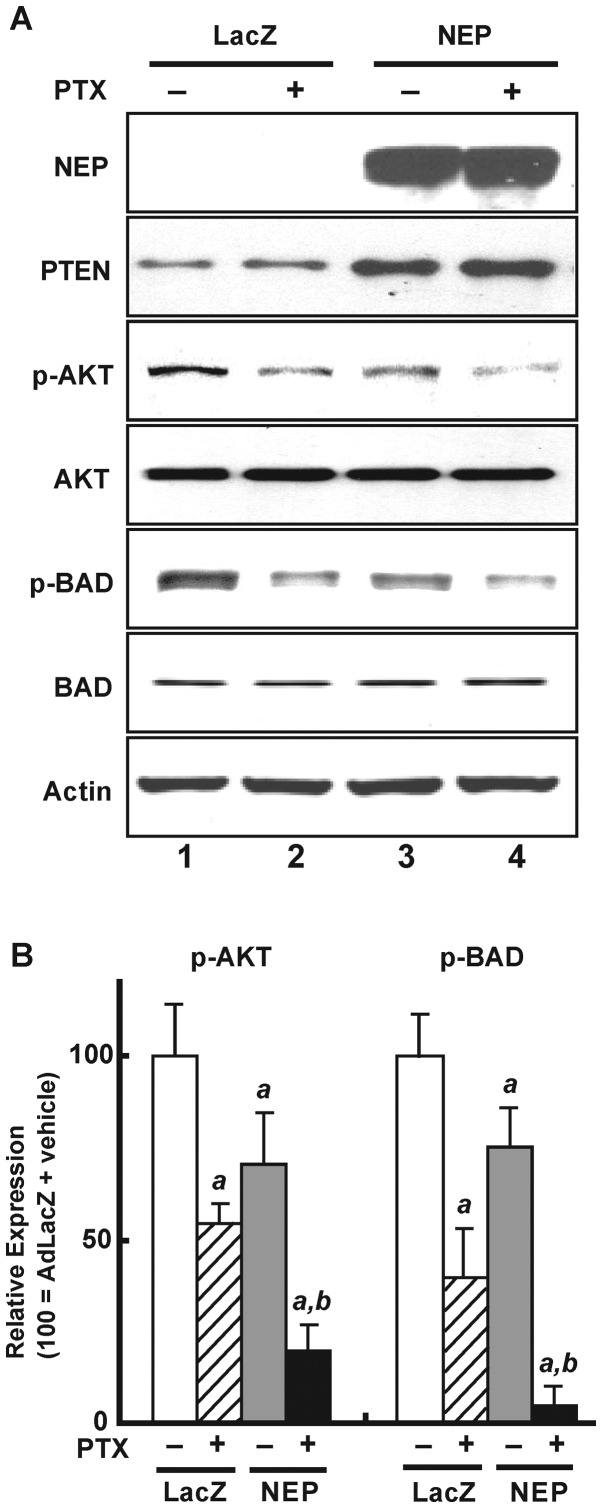
AdNEP infection plus paclitaxel modulates expression of PTEN, phosphorylated Akt and phosphorylated BAD in DU145 cells. (A) Total cell lysates from DU145 cells infected with AdNEP or AdLacZ at 10 pfu/cell treated with paclitaxel (10 ng/ml) (lanes 2 and 4) or vehicle (lanes 1 and 3) were separated by SDS-PAGE and immunoblotted with the indicated antibodies. (B) Schematic representation of expression of phosphorylated Akt and BAD proteins in the different treatment groups. Values are presented as the mean ± SE (n=4). a, P<0.01 compared with AdLacZ plus vehicle treated cells. b, P<0.01 compared with AdLacZ plus paclitaxel treated cells.

NEP stabilizes PTEN protein resulting in decreased phosphor-Akt expression (9). As shown in Fig. 4A, AdNEP infection (rows 3 and 4) leading to NEP expression (first panel) resulted in increased PTEN protein (panel 2, row 3) and decreased Akt phosphorylation (panel 3, row 3) compared with cells infected with AdLacZ (row 1). The addition of paclitaxel to cells infected with AdLacZ (row 2) decreased Akt phosphorylation but did not affect PTEN protein expression. The addition of paclitaxel to cells infected with AdNEP (row 4) significantly further decreased Akt phosphorylation (illustrated in Fig. 4B, left panel) (P<0.02).

We also examined expression of BAD, a pro-apoptotic member of the Bcl-2 family and a substrate of Akt (19). As shown in Fig. 4A (panel 5) and B (right panel), compared with DU145 cells infected with AdLacZ and treated with vehicle (control), BAD phosphorylation significantly decreased in cells infected with AdLacZ treated with paclitaxel (40.4±14.9% of control; P<0.01), in cells infected with AdNEP treated with vehicle (73.8±10.9% of control; P<0.01), and most dramatically in cells infected with AdNEP and treated with paclitaxel (10.2±5.0% of control; P<0.01). Together these results demonstrate that NEP overexpression from adenoviral NEP infection significantly potentiates paclitaxel-induced inactivation of the Akt/Bad signaling pathway.

### AdNEP inhibits DU145 xenograft growth in vivo

**Figure 5:**
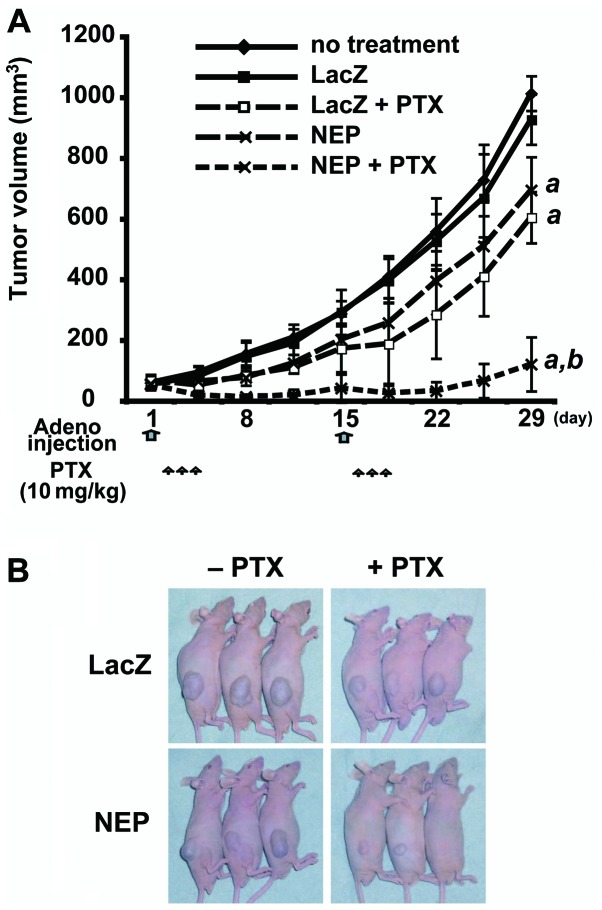
AdNEP infection plus paclitaxel inhibits DU145 xenograft tumor growth. (A) DU145 cells (5×10^6^) were inoculated subcutaneously into the flanks of nude male mice. When tumors reached a size of 4–6 mm in diameter (day 1), they were injected with 100 μl of PBS or PBS containing 1×10^8^ pfu of AdNEP or AdLacZ. On days 2 to 4, mice were injected intraperitoneally with paclitaxel (10 mg/kg). Injections were repeated on day 15 (adenovirus) and days 16 to 18 (paclitaxel). Tumor volumes were measure serially at the indicated times. (B) Representative images of mice containing DU145 xenografts on day 28. Treatments are as indicated and described above.

To evaluate the combined therapeutic efficacy of AdNEP plus paclitaxel *in vivo*, we injected AdNEP or AdLacZ intratumorally on days 1 and 15 into established DU145 xenografts followed by intraperitoneal injection of paclitaxel or saline from days 2 to 4 and from days 16 to 18. At day 28, tumor measurements were performed and the animals sacrificed and the tumors were dissected and weighed. There was no difference between the mean tumor volume of the untreated control cohort (1022.6±255.5 mm^3^) and the AdLacZ plus saline cohort (920.2±238.2 mm^3^). However, the mean tumor volume of the AdNEP plus saline cohort (653.9±230.3 mm^3^, P<0.01) and AdLacZ plus paclitaxel cohort (575.9±176.6 mm^3^, P<0.01) were significantly less (Fig. 5A and B). Furthermore, the tumor volume of the cohort treated with AdNEP plus paclitaxel (122.85±89.5 mm^3^) was markedly less than cohorts treated with AdNEP plus saline or paclitaxel. Similarly, the mean tumor weight of AdNEP plus paclitaxel cohort was significantly less at 28 days than other treatment groups (data not shown). Immunohistochemical staining and for the degree of apoptosis by TUNEL assay of tumors resected at day 6 demonstrated a significant increase in tumors injected with AdNEP and receiving paclitaxel (data not shown). Tumor cells infected with AdNEP showed an increase in NEP protein, an increase in PTEN protein expression and a decrease in p-Akt (data not shown). Phospho-Akt was most diminished in tumors injected with AdNEP and receiving paclitaxel. Together, these results show that in an established prostate cancer tumor xenograft, expression of NEP by injection with AdNEP together with systemic paclitaxel results in an increase in PTEN protein, a decrease in activated Akt, an increase in cell death and inhibition of tumor growth.

## Discussion

The current study was aimed at investigating the potential as therapy for castration resistant prostate cancer of adenoviral delivered NEP in combination with taxane chemotherapy. We have previously demonstrated that recombinant NEP inhibits prostate cancer cell growth *in vitro*(3); overexpression of NEP using a tetracycline repressible system inhibits growth within the mouse prostate in an orthotopic prostate cancer model (8); and injection of Lentiviral-NEP into established xenograft tumors of the CWR22R castration-resistant prostate cancer subline 22RV1 significantly inhibited tumor growth (20). Lentiviral delivered NEP was not effective in inhibiting growth of DU145 cells, although we could demonstrate inhibition of angiogenesis resulting from a decrease in FGF-2 levels as a consequence of NEP catalytic inactivation (20). This lack of effect in DU145 cells presumably resulted in part from the lower level of NEP expression and enzyme activity in lentiviral infected DU145 cells (96.57±10.2 pmol/mg/min) compared to lentiviral infected 22RV1 cells (1171.27 pmol/mg/min). In contrast to lentivirus, infection of DU145 cells with AdNEP in the current study resulted in high levels of NEP protein expression and enzyme activity (962.36±24.5 pmol/mg/min at an infection ratio of 10 pfu/cell). Based on our prior studies of combining recombinant NEP with chemotherapeutic agents, we elected to study the effects of adenoviral delivered NEP in combination with paclitaxel since taxanes are effective in inhibiting prostate cancer growth. We demonstrate that the combination of NEP and paclitaxel significantly and dramatically inhibits Akt activation, DU145 cell growth and tumorigenicity in a mouse model.

There are many potential pathways in which the anti-tumor effects of taxanes and NEP may intersect and augment each other. Akt signaling plays an important role in prostate cancer cell survival and proliferation, as well as chemoresistance (21). NEP negatively effects Akt through multiple mechanisms, including directly interacting with and stabilizing the PTEN protein (22) and catalytic inactivation of neuropeptides, each leading to suppression of Akt phosphorylation.

Studies have also shown that paclitaxel can also inhibit Akt activation and synergize with other agents to induce apoptosis and inhibit cell growth (15,23). We similarly show that paclitaxel treatment results in lower levels of activated Akt which are decreased further by NEP. This is substantiated by increased cleavage of caspase-3 and PARP and inhibition of BAD phosphorylation, resulting in a significant increase in the number of cells undergoing apoptosis. AdNEP augmented the effect of paclitaxel-induced apoptosis through modulation of PTEN/Akt/Bad signaling both in DU145 cells *in vitro* and *in vivo* in xenograft tumors. Our results are similar to a previous report showing that siRNA silencing of Akt enhances the antitumor effects of paclitaxel (24).

Microtubule stabilization through binding of taxane to β-tubulin is the most widely accepted mechanism of taxane’s anti-neoplastic action (25). Once bound by taxanes, microtubules cannot be disassembled and this static polymerization disrupts the normal mitotic process, arrest cells in the G2M cycle phase ultimately leading to apoptosis. Recent studies demonstrate that the cytoskeletal regulatory family of ezrin-radixin-moesin (ERM) can also stabilize microtubule networks (26,27). We and others have shown that the N-terminal domain of NEP directly binds to ERM proteins (10,28,29), and that cells expressing wild-type NEP demonstrate decreased adhesion to hyaluronic acid and cell migration (10). Thus, it is conceivable that via its interaction with ERM proteins, NEP also enhances taxane effects on microtubule stabilization. Finally, the added effect of NEP and paclitaxel may also result from a combined inhibition of protein kinase C (PKC) signaling. PKC isoform PKCδ activity is required for mitochondrial apoptosis in response to etoposide (30) and paclitaxel (31) in various cell types including prostate cancer cells (32,33). We previously reported that NEP stabilizes PKCδ protein expression in prostate cancer cells by inhibiting neuropeptide-induced Src signaling, which in the absence of NEP results in PKCδ protein degradation (34). NEP may augment paclitaxel-induced mitochondrial-dependent apoptosis through increasing PKCδ expression and kinase activity.

The clinical feasibility of adenovirus gene delivery has been demonstrated through intraprostatic injection of adenovirus (35,36). The current study supports the concept of local therapy involving AdNEP injected into recurrent tumor in combination with systemic taxane chemotherapy. The combination of NEP overexpression plus paclitaxel was very effective in inhibiting DU145 tumor growth. This represents a novel therapeutic strategy to target NEP-deficient prostate cancer cells, and would likely be most effective in tumors that have retained PTEN expression. Histone deacetylase inhibition can also increase NEP expression in prostate cancer cells (37), suggesting that HDAC inhibitors may represent another strategy to induce NEP expression. As prostate cancer therapy evolves towards personalized treatments, the approach of identifying NEP deficient tumors for AdNEP replacement therapy in combination with systemic paclitaxel represents a novel approach for treatment.
