# [Corrigendum] Adenoviral neutral endopeptidase gene delivery in combination with paclitaxel for the treatment of prostate cancer

**DOI:** 10.3892/ijo.2024.5694

**Published:** 2024-09-16

**Authors:** Katsuyuki Iida, Rong Zheng, Ruoqian Shen, David M. Nanus

Int J Oncol 41: 1192-1198, 2012; DOI: 10.3892/ijo.2012.1586

Subsequently to the publication of the above article, an interested reader drew to the authors' attention that, for the immunostaining experiments shown in Fig. 3C on p. 1195, the 'NEP' and 'PTX' panels contained overlapping data, such that data which were intended to show the results of differently performed experiments had apparently been derived from the same original source.

After re-examining their original data, the authors have realized that the 'PTX' data panel in Fig. 3C had inadvertently been selected incorrectly. The revised and corrected version of Fig. 3, showing the correct data for the 'PTX' data panel in Fig. 3C, is shown on on the next page. The authors are grateful to the Editor of *International Journal of Oncology* for allowing them this opportunity to publish this Corrigendum, and all the authors agree with its publication. Furthermore, the authors apologize to the readership for any inconvenience caused.

## Figures and Tables

**Figure 3:**
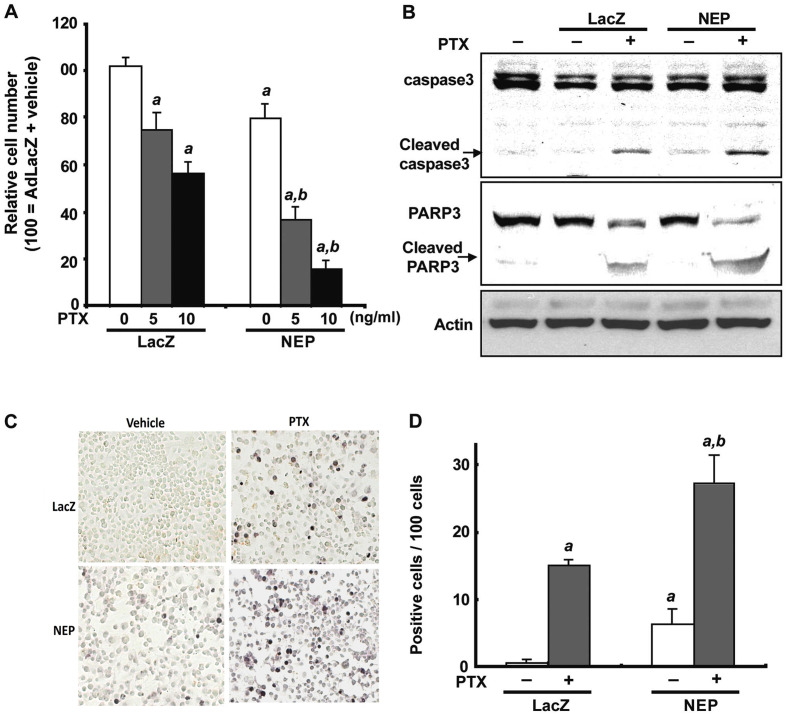
AdNEP infection augments paclitaxel induced apoptosis in DU145 cells. (A) Relative viable cell number of cells infected with AdNEP or AdLacZ in combination with paclitaxel. Following infection with AdNEP or AdLacZ at 10 pfu/cell, cells were treated with paclitaxel at 5 or 10 ng/ml, or vehicle for 48 h and counted at 5 days. Combination therapy with AdNEP plus paclitaxel significantly decreased cell viability. (B) Total cell lysates from DU145 cells treated as indicated and described above were separated by SDS-PAGE and immunoblotted with mAbs to caspase-3 and PARP-1. Infection with AdNEP increased caspase-3 and PARP-1 cleavage products compared with paclitaxel treatment of control cells infected with AdLacZ. (C) Representative immunostain of cells treated with AdLacZ or AdNEP plus paclitaxel. (D) Apoptotic index was calculated as the number of positive cells × 100/total number of cells counted under ×400 magnification in 10 randomly selected areas in each sample. a, P<0.01 compared with AdLacZ plus vehicle treated cells. b, P<0.01 compared with AdLacZ plus paclitaxel treated cells.

